# A Comprehensive Comparison of Tissue Processing Methods for High-Quality MALDI Imaging of Lipids in Reconstructed Human Epidermis

**DOI:** 10.1021/jasms.3c00185

**Published:** 2023-10-16

**Authors:** Maureen Feucherolles, William Le, Jérôme Bour, Carine Jacques, Hélène Duplan, Gilles Frache

**Affiliations:** † Luxembourg Institute of Science and Technology (LIST), Molecular and Thermal Analysis, Materials Research and Technology, L-4422 Belvaux, Luxembourg; ‡ Pierre Fabre Dermo-Cosmétique et Personal Care, Centre R&D Pierre Fabre, Avenue Hubert Curien, 31025 Toulouse Cedex 01, France

**Keywords:** mass spectrometry imaging, skin, reconstructed human epidermis, sample preparation, AP-MALDI, lipids

## Abstract

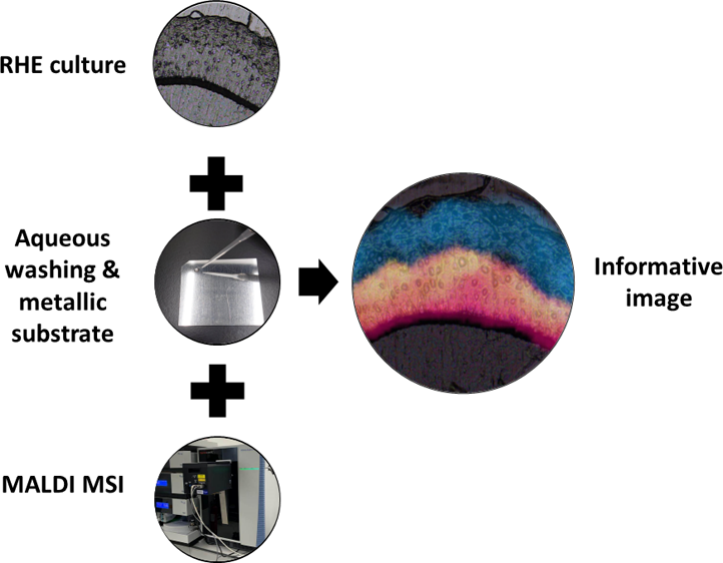

Matrix-assisted laser desorption/ionization (MALDI) mass spectrometry imaging (MSI) has become an important tool for skin analysis, as it allows the simultaneous detection and localization of diverse molecular species within a sample. The use of *in vivo* and *ex vivo* human skin models is costly and presents ethical issues; therefore, reconstructed human epidermis (RHE) models, which mimic the upper part of native human skin, represent a suitable alternative to investigate adverse effects of chemicals applied to the skin. However, there are few publications investigating the feasibility of using MALDI MSI on RHE models. Therefore, the aim of this study was to investigate the effect of sample preparation techniques, i.e., substrate, sample thickness, washing, and matrix recrystallization, on the quality of MALDI MSI for lipids analysis of the SkinEthic RHE model. Images were generated using an atmospheric pressure MALDI source coupled to a high-resolution mass spectrometer with a pixel size of 5 μm. Masses detected in a defined region of interest were analyzed and annotated using the LipostarMSI platform. The results indicated that the combination of (1) coated metallic substrates, such as APTES-coated stainless-steel plates, (2) tissue sections of 6 μm thickness, and (3) aqueous washing before HCCA matrix spraying (without recrystallization), resulted in images with a significant signal intensity as well as numerous *m*/*z* values. This refined methodology using AP-MALDI coupled to a high-resolution mass spectrometer should improve the current sample preparation workflow to evaluate changes in skin composition after application of dermatocosmetics.

## Introduction

The skin is the largest organ of the body and is composed of three different layers, namely, the epidermis, dermis, and subcutaneous tissue. It is exposed, both acutely and chronically, to a wide variety of xenobiotics. The major function of the skin, due to its main barrier, the *stratum corneum*, is to prevent water loss and protect against environmental hazards, such as bacteria, chemicals, and sun exposure.^1^ In addition to its primary role as a barrier, skin is a metabolically active tissue that contains enzymes capable of metabolizing not only endogenous chemicals but also xenobiotics. Skin diseases, such as dermatitis or psoriasis, are not uncommon and are reported to be the fourth leading cause of nonfatal morbidity worldwide since 2010, with 41.6 million disability-adjusted life years in 2013.^2,3^ Recent advances in skin biology research have increased the understanding of many skin functions and mechanisms involved in skin regeneration,^4^ immunity, and inflammation.^5^ Additionally, the importance of lipids in skin disease pathogenesis has been brought to the forefront of investigations. For example, lipids belonging to the glycerophospholipids class have been extensively explored for their role in inflammatory process.^6,7^ While conventional mass spectrometry (MS) methods, such as liquid chromatography (LC)-MS/MS, measure skin composition, they do not provide information on their spatial structure. Conversely, immunofluorescence and other conventional targeted histological staining methods can localize certain molecules of interest but are not as selective as the MS methods. Therefore, the development of new untargeted technologies which enable a better molecular understanding and localization of skin components are of great importance.^8^

Matrix-assisted laser desorption/ionization (MALDI) mass spectrometry imaging (MSI) has grown in popularity over the last two decades due to its label-free detection of a large range of biomolecules, such as small metabolites, lipids, peptides, and proteins, in complex samples. In contrast to targeted imaging methods, such as immunohistochemistry, MALDI-MSI is a valuable method for investigating both the composition and spatial distribution of diverse molecular species in a sample, providing insights into biological systems.^9^ Basic principles of the method are extensively detailed elsewhere.^10,11^ Over the past decade, MALDI MSI has become an important tool for analyzing skin, with numerous reports supporting its application in the concurrent determination of lipids composition and their related distribution in skin samples.^12−15^ For example, De Macedo et al. compiled a list of lipids and their distribution in the skin of leprosy patients before and after multidrug therapy.^12^ Ellis et al. added the structural identity of almost every lipid ion detected by an automated MS/MS acquisition method to the list of known skin components.^13^ Furthermore, MALDI MSI has also been used to evaluate the penetration of different formulations, including photoprotective products, creams, and balms.^16−18^ For example, Jacques et al. compared the skin penetration and localization of TriAsorB formulated in a new SPF50+ photoprotective system with three other sunscreens by combining MALDI MSI and ToF SIMS.^17^ Early studies applied MALDI MSI to provide a qualitative assessment, but since then, it has become possible to also use it to provide simultaneous information on the distribution and quantity of compounds in the skin.^19−21^ For example, Traberg et al. used a skin mimetic tissue model with a reproducible and defined composition to develop an imaging method to quantify bleomycin in skin. The developed MSI workflow resulted in tissue concentrations similar to those measured using LC-MS.

Traditionally, *in vivo* animal models and *ex vivo* animal skin have been used for measuring skin penetration and local skin toxicity, with the obvious advantage that these are easy to obtain; however, they have several drawbacks.^22^ These include high costs, ethical issues, and structural differences between animal and human skin.^22,23^ An example of the latter is rabbit skin, which may not be the best model for investigating human skin scarring, since it has an additional cartilage layer compared to human skin.^23^ In parallel with these limitations, there is a growing global trend to reduce or replace animal testing by *in vitro* testing. This is especially relevant to the cosmetics industry due to the full ban on animal testing for cosmetic ingredients, which came into force in the European Union on March 2013 (EU Regulation 1223/2009). For this reason, *ex vivo* human skin models are the best surrogates for *in vivo* human studies, although they too have limitations regarding costs and a regular supply of tissue. Therefore, there has been an increased need to develop and validate alternatives to animal skin and *ex vivo* human models. Since the 90s, several artificial several skin models, so-called reconstructed human epidermis (RHE) models, have been developed, including commercial solutions, such as EpiSkin,^24^ KeraSkin,^25^ SkinEthic,^26^ and EpiDerm,^27^ or models derived using an open-source protocol.^28^ We have focused on the SkinEthic model, whereby human keratinocytes are cultured on an inert polycarbonate filter at the air–liquid interface. These RHE models mimic many characteristics of the upper layers of native human skin, i.e., the epidermis, including morphology, lipid composition, as well as biochemical markers.^29,30^ Therefore, they can be used to evaluate adverse effects of chemicals present in simple or complex formulations applied to the skin such as irritation, corrosion, or UV exposure testing. The use of RHE models for toxicological studies has increased considerably over the last two decades due to their reproducibility.^31,32^ This attribute of *in vitro* skin models was important for their use in the development of guidelines by the Organization for Economic Co-operation and Development (OECD) to replace *in vivo* animal methods to measure local skin effects. As a result, OECD test guidelines for skin irritation, previously evaluated using animal methods only (OECD TG 404), now include two *in vitro* assays using RHE models (OECD TG 431 and OECD TG 439).^33−35^

There are currently only a few publications describing the feasibility of combining the use of MALDI MSI and *in vitro* skin models.^36−38^ Most reports investigated sample preparations from *ex vivo* human or experimental animal skin.^8,39^ Moreover, there is only one publication using both MALDI MSI and an RHE model.^40^ Indeed, the major challenge of the RHE model MALDI MSI compared to *ex vivo* skin is the thickness of the sample. For the RHE model, the thickness is around 100 μm, which comprises the *stratum corneum* and viable epidermis. *Ex vivo* skin samples comprised dermis, which increase the thickness of the skin sample up to 1000 μm. In this study, the authors analyzed the absorption and distribution of an antidepressant drug into the Straticell-RHE-EPI/001 model. Skin samples were cut into 5 μm tissue sections, mounted on aluminum plates, and sprayed with a matrix of α-cyano-4-hydroxycinnamic acid matrix (HCCA) before being analyzed at a spatial resolution of 150 μm. Information on the optimization of the sample preparation workflow for *in vitro* RHE models is currently lacking, and it should not be assumed that conditions that are optimal for native human or animal skin are also optimal for RHE models. In addition, now that specific hardware can reach a pixel size averaging 5 μm with high mass resolution and high sensitivity, it is possible to improve the ability to locate numerous metabolites within a tissue section. Therefore, the aim of this study was to investigate the effect of RHE model sample preparation techniques on the quality of lipid analysis using MALDI MSI. To this end, we evaluated the impact of different substrates, sample thickness, and tissue treatments, including washing and recrystallization for fixed matrix parameters.

## Materials and Methods

### Tissue Samples: RHE Culture

**Figure 1:**
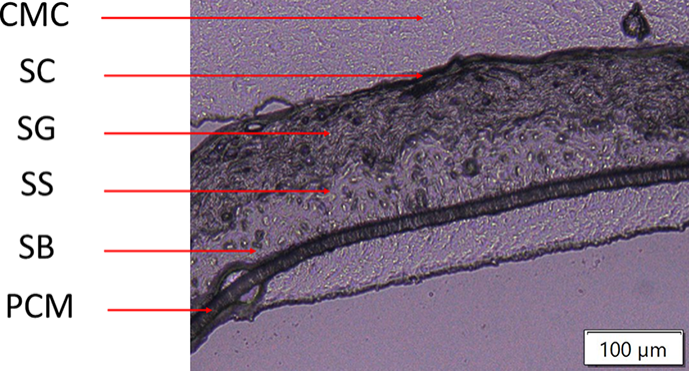
EPISKIN reconstructed human epidermis (SkinEthic) used in the impact study. CMC: Carboxymethylcellulose, SC: *Stratum corneum*, SG: *Stratum granulosum*, SS: *Stratum spinosum*, SB: *Stratum basale*, PCM: Polycarbonate membrane. Bar scale: 100 μm.

SkinEthic RHE models (Figure 1) at day 17 (3 replicates per condition; 0.5 cm^2^) were placed into wells containing SkinEthic medium (EPISKIN) at 37 °C in a 5% CO_2_ air incubator and stabilized for 4 h. The RHE models were exposed to solar simulated radiation (SSR) at a dose of 16.5 J/cm^2^ using a Suntest Heraus Instrument CPS+ instrument (2MED: minimal erythemal dose). After irradiation, RHE models were incubated for 24 h.

### Embedded Frozen Tissue and Cryo-Sectioning

RHE models were embedded in a mixture of 10% gelatin and 2.5% carboxymethylcellulose (CMC) diluted in water. Embedded RHE models were frozen in 2-methylbutane and liquid nitrogen and stored at −80 °C until analysis. To evaluate the impact of tissue section thickness for MALDI MSI, samples were sliced into sections of different thicknesses, i.e., 10, 6, 5, and 4 μm using a Cryo-Ultramicrotome Leica EM FC6 (Leica Microsystems GmbH, Germany) set at −20 °C and thaw-mounted onto different substrates. The following substrates were selected based on materials used in the literature and their related resistivity (Table 1): conventional microscope glass slides,^36^ indium tin oxide (ITO)-coated glass slides,^41^ aluminum plates,^38,40^ stainless-steel plates, and stainless-steel plates coated with 3-aminopropryltriethoxsilane (APTES, commercially available under the name VECTABOND reagent) as an adhesion promoter for glass slide.^42^

**Table 1:** Resistivity and Conductivity of Tested Substrates

Substrate	Electrical Resistivity (Ω·cm)	Electrical Conductivity (S·cm)
Glass	1 × 10^15^	1 × 10^–15^
Glass + ITO	26.2	3.8 × 10^–2^
Aluminum	0.3	3.3
Stainless steel	0.2	5
Stainless steel + APTES	0.2	5

To confirm the APTES coating was complete, time-of-flight secondary ion mass spectrometry (TOF-SIMS) analysis was performed (Supplementary File 1). The electrical resistivity in ohm centimer (Ω·cm) was measured using a multimeter, and electrical conductivity in siemens centimeter (S·cm) was calculated using an online conversion software (https://www.cactus2000.de/uk/unit/masscnd.php). Tissue samples were kept at −80 °C until analysis.

### Sample Preparation

Optical images of the tissue sections were captured using an Olympus BX51 Microscope (Olympus, Belgium). Once the optimal substrate and thickness were established, air-dried tissue sections were either left untreated or washed with ammonium acetate (NH_4_Ac, 50 mM, pH6.5) or with distilled water chilled to 4 °C, by pipetting three times for 5 s.^14,43^ RHE tissues were coated with 24 layers of HCCA (3 mg/mL in acetonitrile:H_2_O 1:1 solution + 0.2% trifluoroacetic acid) using a SunCollect MALDI-Sprayer (SunChrom GmbH, Germany) at a flow rate of 15 μL/min at a velocity of 600 mm/min, on a Z axis position of 25 mm. The HCCA matrix was selected, as it was recommended for high spatial resolution imaging in the positive-ion mode.^44^ The impact of a matrix recrystallization based on 5% propan-2-ol (IPA) to improve lipid ion signals was also evaluated.^45^ As described by Dueñas et al., samples were placed in the recrystallization chamber, in which a filter paper was soaked with 1 mL of 5% IPA for 2 min at 55 °C. The excess solvent was removed by evaporating it for 2 min at 55 °C outside the recrystallization chamber.

### MALDI Imaging and Data Processing

MALDI analysis was performed using an AP-MALDI UHR ion source (Masstech Inc., USA) coupled to an Exploris 480 high-resolution mass spectrometer (Thermo-Fisher Scientific, USA) in positive ion mode, combined with the EASY-IC source to produce an in-spectrum lock mass for scan-to-scan mass scale recalibration. For imaging, the ion source was operated in a “Constant Speed Raster” motion mode with a spatial resolution of 5 μm per pixel. The laser was operated at a frequency of 400 Hz with 3% laser energy. Spectra were acquired with a 490 ms injection time, over a mass range of 205–2000 Da and at a mass resolution of 240 000 @ *m*/*z* 200. The automatic gain control (AGC), used for controlled injection of the number of ions, was disabled to ensure an equal injection time for all pixels. Concomitant with the RHE image acquisition, small images of the embedding medium (carboxymethylcellulose/gelatin, i.e., CMC) and substrates were acquired as a blank for background peak measurements. Raw image files were converted into imzML and imported into LipostarMSI software (v.1.3.1b) (Molecular Horizons Srl, Italy)^46^ for image processing and molecular identification (±5 ppm tolerance) based on the Lipid Maps^47^ structure database. To identify the peaks in LipostarMSI, the mass score and the isotopic pattern score were used as metrics. The mass score is based on the proximity of the experimental mass to the theoretical mass of the proposed database match. The isotopic pattern score is a comparison of the experimental isotopic pattern abundance and spacing with the corresponding theoretical attributes of the proposed match. The resulting images were not normalized to the total ion current (TIC) to observe any intensity differences of *m*/*z* values due to the different treatments. Additional data visualization was performed using R 4.2.2 (ggVenn and UpSetR packages^48^) and Python 3.9.12 (Seaborn, Matplotlib, and Pandas libraries) software.

## Results

### Impact of the Substrate

In a first step, untreated RHE tissues mounted on different substrates, i.e., conventional microscope glass slides, ITO-coated glass slides, aluminum plates, or stainless-steel plates with and without APTES, were analyzed to evaluate the impact of substrate on the ion signal intensity. To track the suitability of each substrate, two biomarker *m*/*z* values were used, namely 414.4304 and 760.5847. The ion at 414.4304 *m*/*z* is a marker for the upper part of the epidermis, including the *stratum corneum* and *granulosum*.^49^ The ion at 760.5847 *m*/*z* is a marker for the lower part of the epidermis, including the *stratum basale* and *stratum spinosum*. Based on their molecular formulas, the two biomarkers were identified as a fatty acyl compound (SFE (26:0), [M + NH_4_]^+^, mass score: 98.77%, isotopic pattern score: 94.48%, mass delta: 0.4 ppm) and phosphatidylcholines (PC (34:1), [M + H]^+^, mass score: 98.37%, isotopic pattern score: 97.98%, mass delta: 0.5 ppm), respectively.

**Figure 2:**
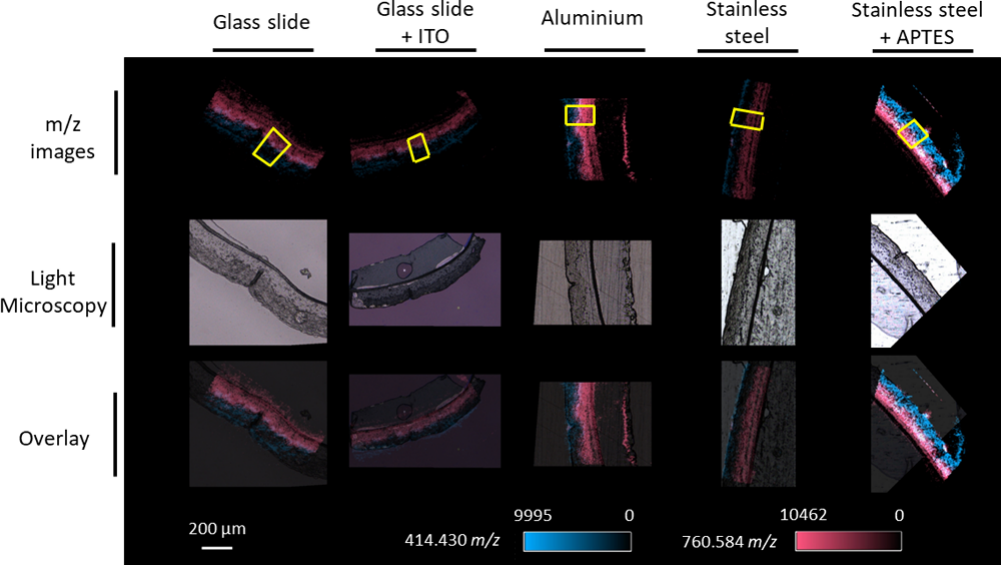
Effect of the substrate on the ion signal of the 414.4304 (SFE (26:0), [M + NH_4_]^+^, blue) and 760.5847 *m*/*z* (PC (34:1), [M + H]^+^, red) values across nontreated RHE tissue sections (10 μm thick) analyzed by MALDI MSI at 5 μm spatial resolution. Yellow boxes represent the region of interest (ROI) used for averaging mass spectra.

**Figure 3:**
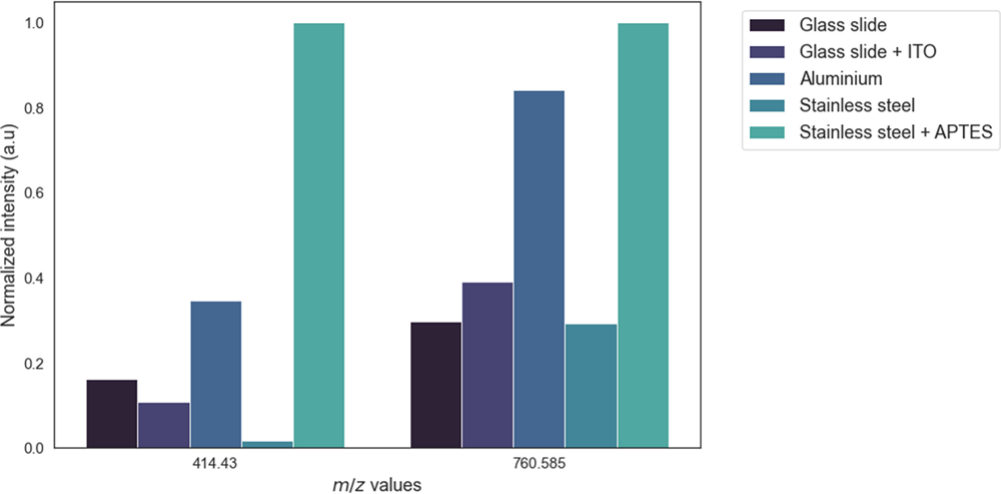
Region of interest (ROI) averaged mass spectra for the 760.5847 and 414.4304 *m*/*z* values in RHE tissue sections mounted on different substrates.

The two biomarkers were overlaid using the two *m*/*z* images (Figure 2). The intensity signals of the biomarkers were low in RHE models mounted on glass slides with or without an ITO coat as well as on stainless-steel plates but were more intense in RHE models fixed on aluminum plates and stainless-steel plates coated with APTES. Mass spectra of pixels in the region of interest (ROI), including all epidermis layers, were averaged to quantify the trend observed qualitatively according to the imaging (Figure 3). This confirmed that the use of aluminum plates and APTES-coated stainless-steel plates produced the highest intensity signal, whereas the intensity was lower when glass slides were used. Interestingly, the intensity signal for the upper part of the epidermis of RHE models mounted on regular stainless-steel plates was lower than that on other substrates with higher or lower conductivities (Table 1).

**Figure 4:**
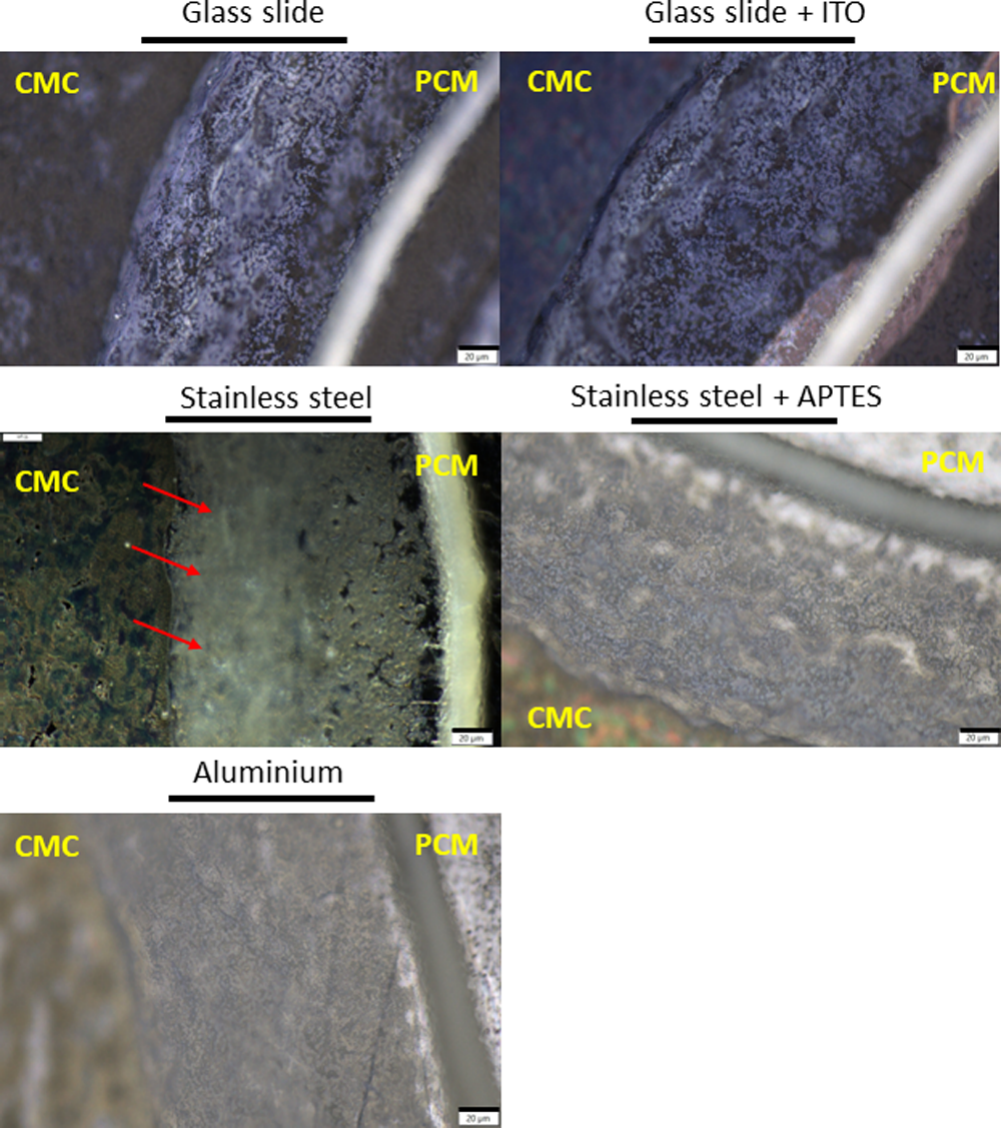
Optical microscopy of RHE tissue sections (10 μm thick) on different substrates after matrix spray application. Bar scale: 20 μm. Red arrows indicate that the *stratum corneum* is out of focus. CMC: Carboxymethylcellulose, PCM: Polycarbonate membrane.

When observed under a light microscope, the extent of tissue adhesion was different depending on the type of substrate used (Figure 4). When a tissue becomes detached from the substrate, the locations of the cell layers are on different planes of focus. For most substrates, such as glass or aluminum, the different cell layers of a well-attached tissue section are on the same topographic plane. However, this was not the case for RHE tissue sections on a stainless-steel substrate, whereby the *stratum basale* and *stratum spinosum* were on the same plane, while the *stratum corneum* and *granulosum* appeared to be on a higher plane. This configuration could be the reason for the limited intensity of the ion signal measured at 414.4304 *m*/*z* (i.e., the biomarker for the outer layers of the RHE) for tissues on the stainless-steel substrate. The APTES coating helped to improve adhesion to the stainless-steel substrate and, consequently, the signal intensity. Therefore, since APTES-covered stainless-steel plates resulted in the best adhesion and intensity signals for both biomarkers, these were used for the rest of the study.

### Impact of the Thickness of the Tissue

**Figure 5:**
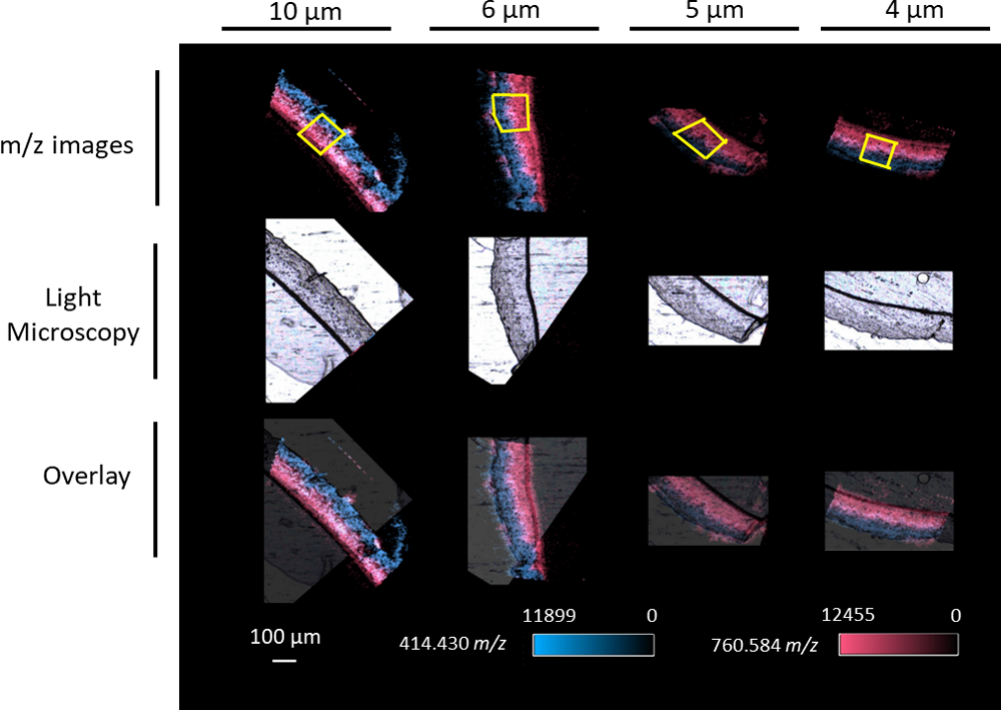
Example of imaging of RHE tissue sections of different thicknesses. Blue: 414.4304 *m*/*z* (SFE (26:0), [M + NH_4_]^+^) and red: 760.5847 *m*/*z* (PC (34:1), [M + H]^+^). Yellow boxes represent the region of interest (ROI) used for averaging mass spectra.

**Figure 6:**
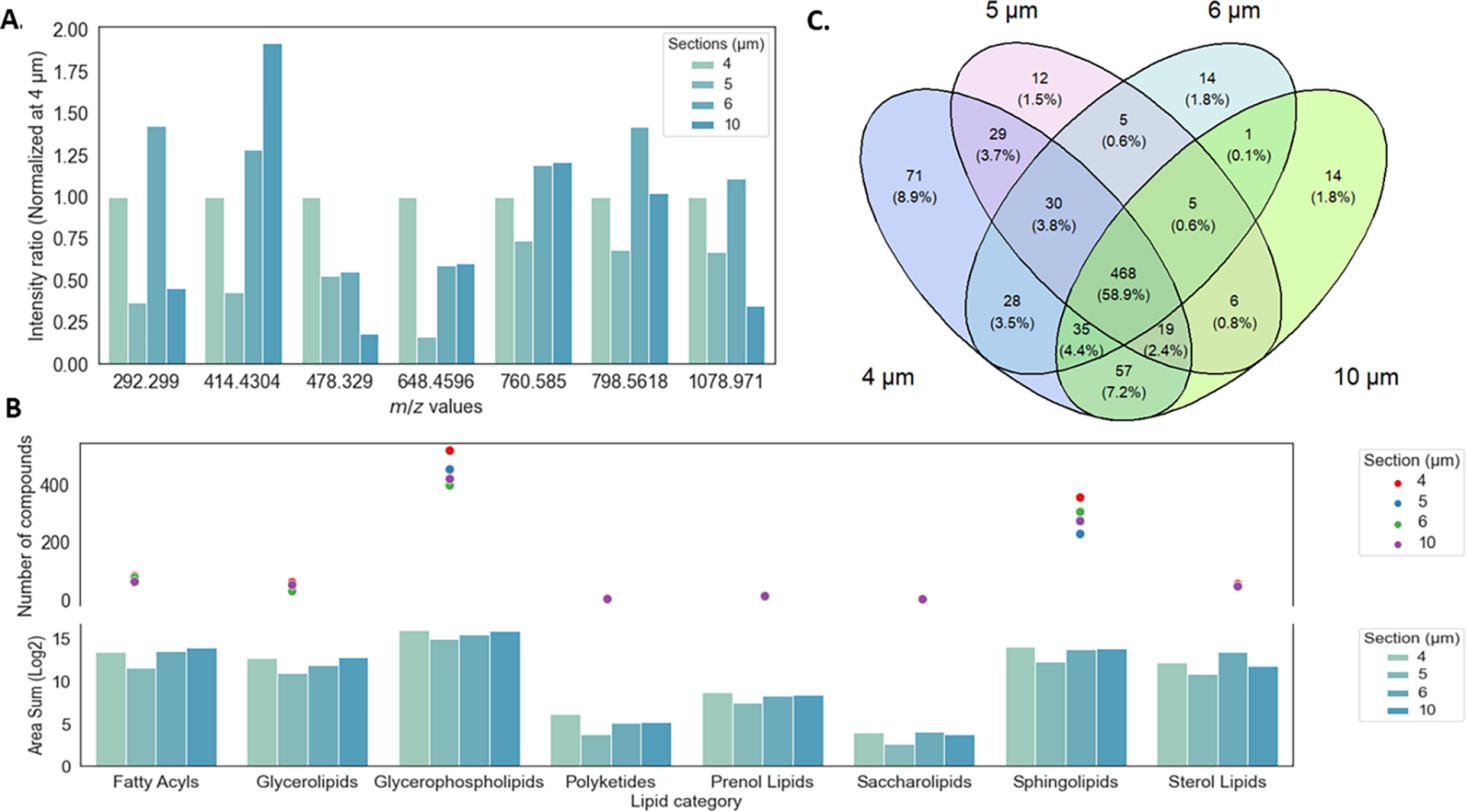
Effect of the RHE tissue section thickness on averaged intensity ratio (normalized at 4 μm) on *m*/*z* values of the studied range (A) and on lipid categories changed and the number of lipid compounds detected within selected ROI with a mass score >85% (B). Venn diagram showing the relationship between unique compounds identified in the four tissue section thicknesses (C). A total of 737, 574, 586, and 604 unique compounds were detected in 4, 5, 6, and 10 μm thickness, respectively.

Different thicknesses of RHE tissues, i.e., 10, 6, 5, and 4 μm, were mounted on APTES-coated stainless-steel plates to evaluate the impact of the tissue section thickness on the ion signal intensity and the number of lipid categories and lipids identified. Figure 5 shows the two target biomarkers overlaid for the different RHE tissue section thicknesses. Sections of 10 and 6 μm thickness appeared to result in the highest signal intensity for 414.4304 and 760.5847 *m*/*z*. However, when the effect of the RHE model thickness on the ROI averaged intensity ratio (normalized to 4 μm) was investigated for other mass ranges (Figure 6A), no clear trend identified. Indeed, while the 10 μm thickness resulted in the most intense signals for the two target biomarkers, it did not result in the highest intensity of other *m*/*z* values representing other common skin biomarkers present in the *m*/*z* range measured, e.g., 292.299 *m*/*z* (eicosatetraene, [M + NH_4_]^+^), 478.329 *m*/*z* (ecalcidene, [M + Na]^+^), 1078.971 *m*/*z* (omega-linoleoyloxy-Cer(t18:1(6OH)/32:0, [M + Na]^+^). A thickness of 4 μm resulted in a particularly good intensity of 478.329 *m*/*z* and 648.4596 *m*/*z* compared to other thicknesses as well as a generally good intensity of most *m*/*z* values evaluated.

As the RHE thickness might have a direct impact on the ion signal intensity, it might also impact the number of detected peaks and, hence, lipids categories. Therefore, the number of monoisotopic values detected within the ROI sections were analyzed. A total of 7706 monoisotopic *m*/*z* values, including 2419 matches with the LipidMaps database, were detected across all MS images. However, many of these values were outside of the tissue signal. Therefore, the background peak list (*n* = 15 815 monoisotopic *m*/*z* values detected in matrix-coated embedding medium or substrate, acquired in parallel to the RHE tissue section images) was used to clean up the peak list from the tissue area. This resulted in 1487 entries with a hit a match with the LipidMaps database, regardless of the mass and isotopic pattern scores (Supplementary File 2). When the identification was refined to include only mass scores greater than 85%, 1143 *m*/*z* values matched an entry of the database, including 160 *m*/*z* values with an isotopic pattern score ranging from 33 to 97%. Overall, seven main categories of lipids were identified in the different model sections, fatty acyl, glycerolipids, glycerophospholipids, sphingolipids, sterol lipids, prenol lipids, saccharolipids, and polyketides (Figure 6B). Based on the area sum plot, there were no differences between RHE tissue section thicknesses observed for each of the lipid categories. However, the number of compounds identified per tissue section thickness was different. Totals of 1089, 839, 867, and 869 compounds were identified in tissue sections with thicknesses of 4, 5, 6, and 10 μm, respectively. Interestingly, while the number of compounds representing fatty acyls, glycerolipids, polyketides, prenol lipids, saccharolipids, and sterol lipids was similar across the tissue section thicknesses, the number differed for glycerophospholipids and sphingolipids. Indeed, 520, 454, 398, and 421 *m*/*z* values were identified as glycerophospholipids, with 323, 279, 243, and 261 unique matches, for tissue sections with a thickness of 4, 5, 6, and 10 μm, respectively. Likewise, 356, 229, 306, and 274 *m*/*z* values were identified as glycerophospholipids, with 223, 155, 195, and 185 unique matches, for tissue sections with a thickness of 4, 5, 6, and 10 μm, respectively.

Interestingly, 71 specific unique compounds were identified in the ROI of the 4 μm tissue sections (Figure 6C, Supplementary File 2), including 6 fatty acyls, 7 glycerolipids, 33 glycerophospholipids, 2 polyketides, 20 sphingolipids, and 3 sterol lipids.

When selecting the best conditions, the practical aspects of obtaining the samples should be considered. While 4 μm RHE tissue sections were optimal with respect to the signal intensity of most of the *m*/*z* values and the number of compounds representing glycerophospholipids and sphingolipids, they are difficult to handle during cryo-sectioning and thaw-mounting. Indeed, a significant number of 4 μm RHE tissue sections were torn, flipped, or twisted, making them unusable for further MSI analyses. As a note, it has been observed that finely cut sections of RHE, e.g., 4 μm, show better adhesion to steel substrates than those at 10 μm (data not shown). Therefore, we selected a thickness of 6 μm as a reasonable compromise between technical difficulty and achieving a sufficient intensity ratio (which was similar to that in 4 and 10 μm thick sections) and the number of identified compounds.

### Effect of the Pretreatment on Samples

In a final step, different types of tissue pretreatments were evaluated. These included a washing step with either NH_4_Ac or Milli-Q water and a recrystallization step with 5% IPA.

**Figure 7:**
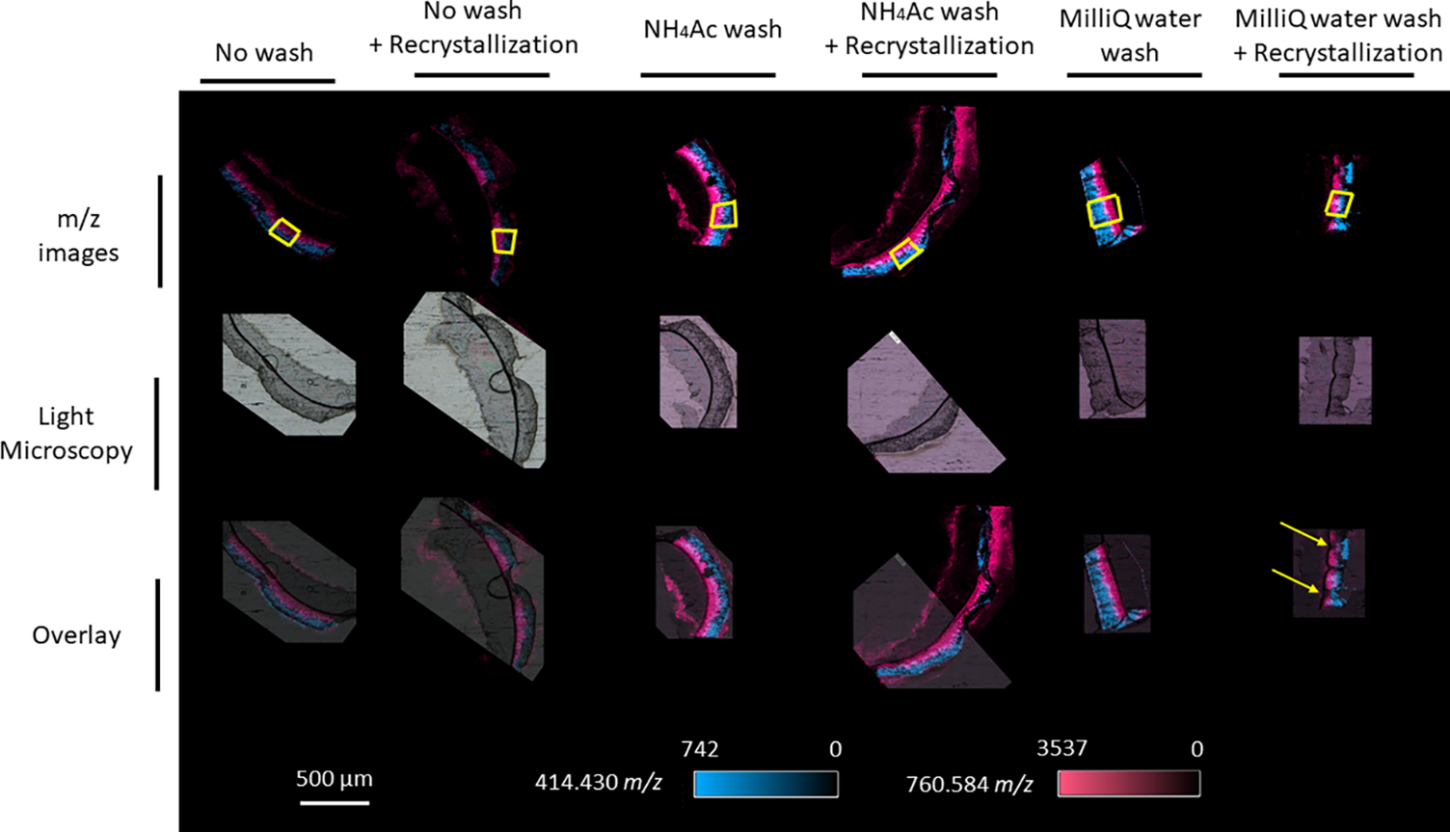
Example of RHE imaging of RHE models with different pretreatments. Blue: 414.4304 *m*/*z* (SFE (26:0), [M + NH_4_]^+^) and red: 760.5847 *m*/*z* (PC (34:1), [M + H]^+^). Yellow boxes represent the region of interest (ROI) used for averaging the mass spectra. The yellow arrows indicate twisted tissue sections after recrystallization.

**Figure 8:**
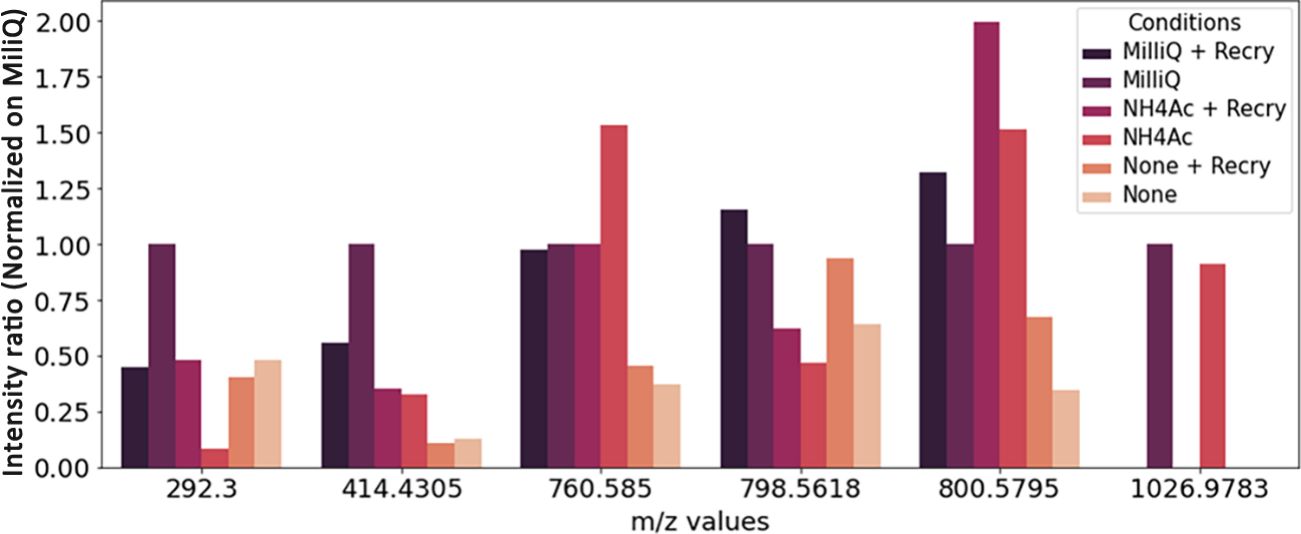
Effect of pretreatment on the intensity ratio based on ROI averaged mass spectra for different *m*/*z* values for 6 μm thick RHE tissue sections mounted on APTES-coated stainless-steel plates.

Biomarkers for the upper and lower epidermis layers, i.e., 760.5847 and 414.4304 *m*/*z* respectively, were overlaid on the different optical microscope images (Figure 7). Both washing methods significantly increased the signals of the *m*/*z* values compared to those of unwashed tissue sections. However, recrystallization alone improved the signal of washed and unwashed samples. Importantly, the recrystallization step could move and twist the tissue section if it is poorly attached to the substrate, which was the case for tissue sections washed with Milli-Q water (Figure 7) and other tissue sections (Supplementary File 3). When the two types of washing steps were compared based on the intensity ratios of several *m*/*z* values, washing with Milli-Q water resulted in higher ratios than with NH_4_Ac for certain *m*/*z* values (Figure 8). For example, for 414.4305 *m*/*z*, the signal intensity after washing with Milli-Q water was 3-fold higher than that using NH_4_Ac. By contrast, the intensity signal of 760.5847 *m*/*z* was highest after washing with NH_4_Ac. Interestingly, the impact of recrystallization depended on the *m*/*z* of interest, whereby they were unaffected, decreased, or even abolished; however, recrystallization tended not to increase the intensity signal.

Since washing and recrystallization steps had positive and negative impacts on the ion signal intensity, respectively, they could also affect the number of detected peaks and, hence, lipids categories. Therefore, the number of monoisotopic values detected within the ROI sections was analyzed (Figure 7). A total of 30 855 monoisotopic *m*/*z* values, including 5980 matches with the LipidMaps database (based on molecular formula), were detected across all ROIs. However, many of these values were outside tissue signal. Therefore, based on the blank peaks list (*n* = 17 120 monoisotopic *m*/*z* values) present in the RHE tissue section images acquired in parallel, the latter were removed from the selection. This resulted in 750 entries with a match with the LipidMaps database, regardless of the mass score and isotopic pattern score (Supplementary File 4). By refining the identification to include only entries with a mass score greater than 85%, there were 637 *m*/*z* values which matched an entry of the database, including 234 *m*/*z* values with an isotopic pattern score ranging from 33 to 99%.

**Figure 9:**
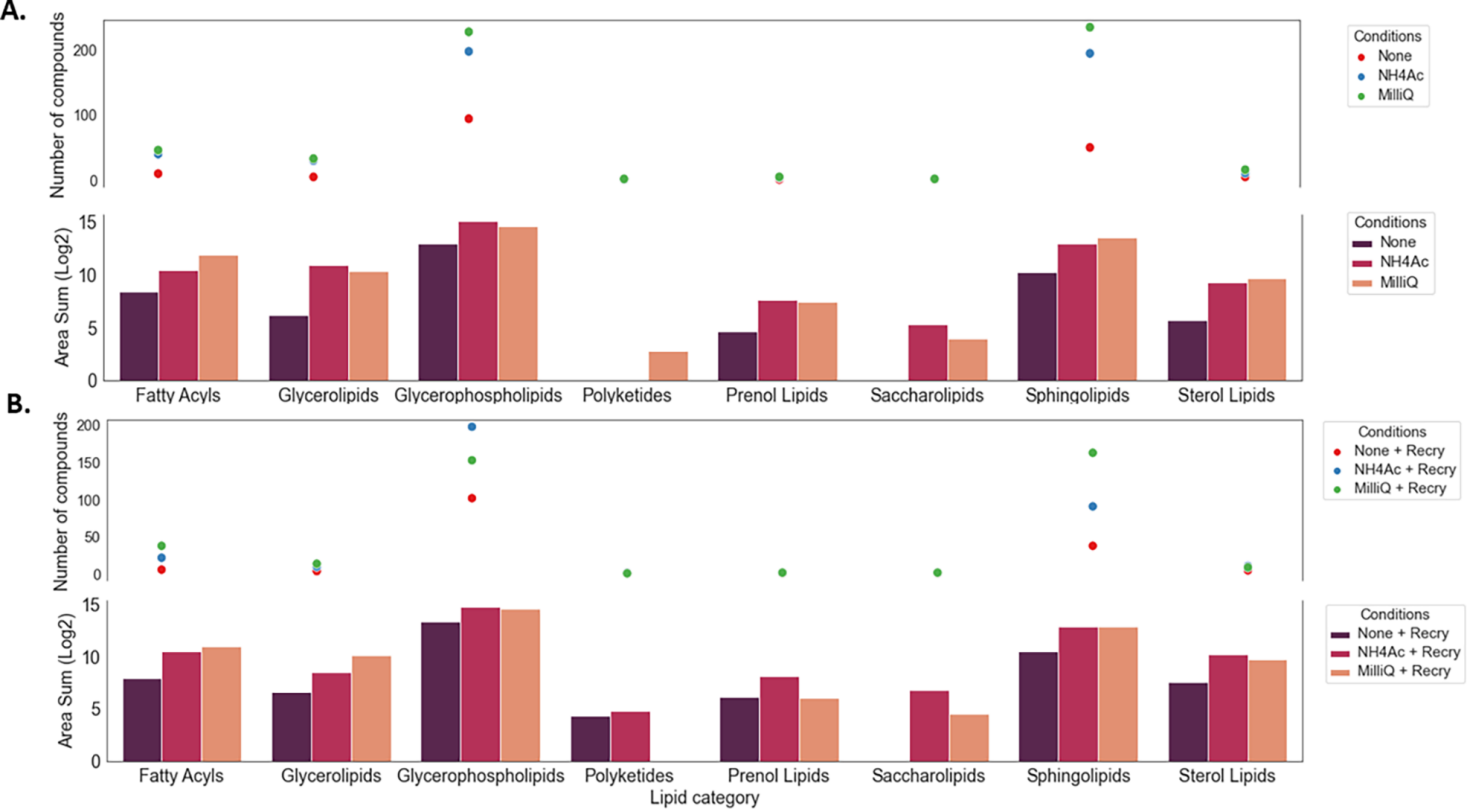
Impact of the pretreatment with washing (A) and recrystallization (B) on lipid category changes and the number of lipid compounds detected within a selected ROI with a mass score >85%.

Meanwhile, the seven main categories of lipids were identified in RHE tissue sections washed with Milli-Q water (Figure 9A); polyketides were not detected in unwashed tissue sections or tissue sections washed with NH_4_Ac. Also, saccharolipids were not detected in nonwashed tissues. Washed RHE tissue sections generally exhibited a higher area sum than unwashed tissue sections. Washed samples also had more compounds per lipid category than unwashed samples. For example, there was a total of 226, 196, and 93 *m*/*z* values representing glycerophospholipids identified in tissue sections washed with Milli-Q water or NH_4_Ac, or left unwashed, respectively. Based on these findings, it was concluded that the sample preparation should include washing with Milli-Q water only.

**Figure 10:**
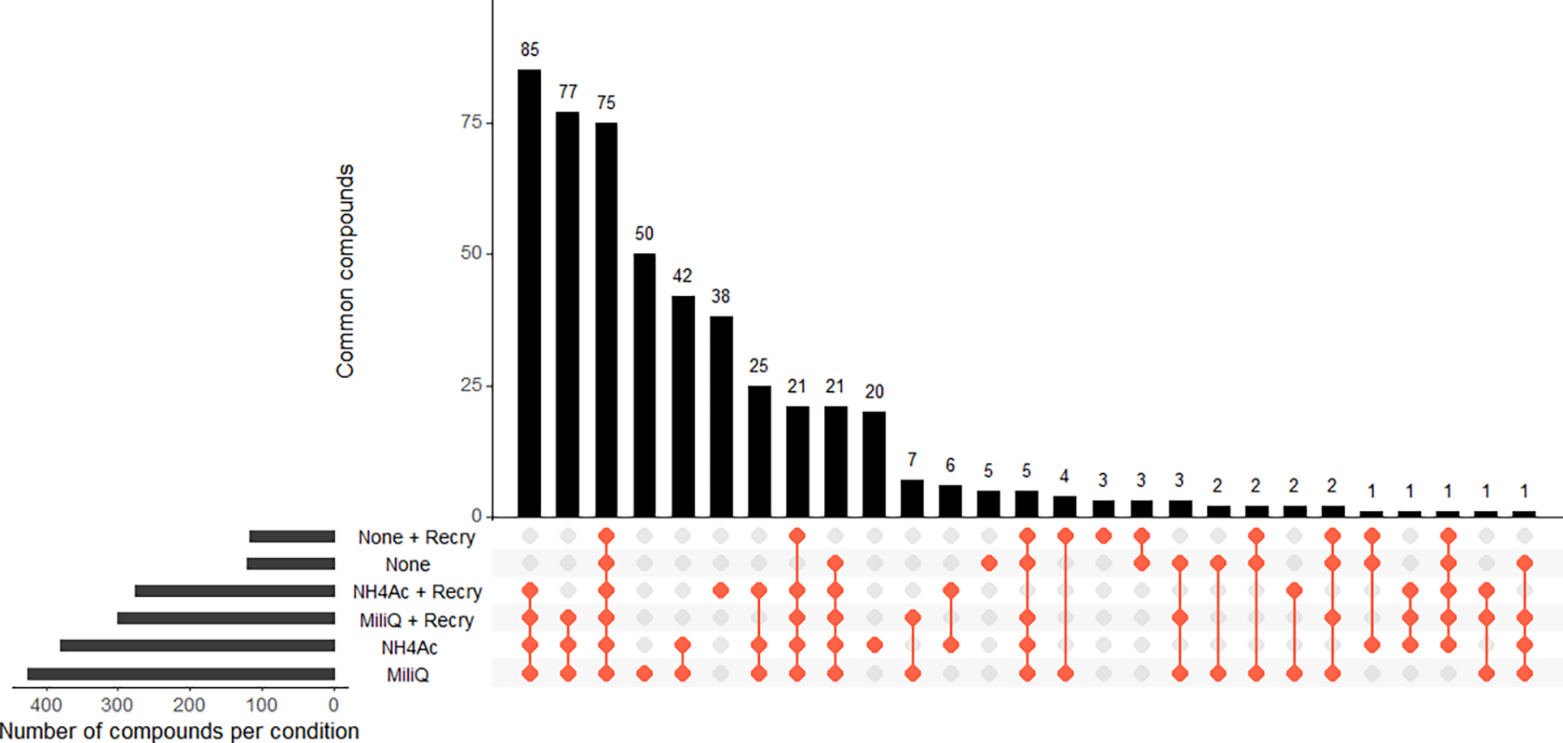
UpSet plot of the number of common and specific lipid compounds across the different pretreatment conditions.

Recrystallization did not provide any additional improvement in the organization of lipid categories (Figure 9B); moreover, the number of compounds identified after recrystallization was decreased. There were 226 and 233 *m*/*z* values identified as glycerophospholipids and sphingolipids, respectively, before recrystallization and only 152 and 162 *m*/*z* values, respectively, after recrystallization. Furthermore, while most of the lipid compounds were detected in washed tissue, only 50 and 42 specific compounds were detected in tissues washed with Milli-Q water alone or in both solutions, respectively (Figure 10). Due to its detrimental effects, the optimal sample workflow should not include recrystallization after washing.

## Discussion

A major advantage of MALDI MSI of biological tissues is that it preserves the integrity of the sample so that the spatial distribution and abundance of biomolecules can be determined in conditions close to the native state.^50^ Recent investigations on the influence of hardware parameters, such as the impact of the wavelength on biological nonflat samples^51^ or the impact of the laser spot size on lipid signals from brain sections,^52^ have led to the improvement of the signal intensity and the ability to image at cellular and subcellular levels.^53^ In addition to these aspects, it is also essential to optimize the sample preparation to increase the number of biomolecules detected and the ion signal intensity. General and analyte-specific aspects of skin sample preparation, including washing and matrices and their deposition, have already been discussed extensively by de Macedo et al.^8^ However, to the best of our knowledge, there is no report investigating the impact of the substrate, sample thickness, and matrix recrystallization of RHE models. Based on a specific HCCA coating protocol, optimized to ensure minimal delocalization at 5 μm lateral resolution, the main findings from this study are that (1) a stainless-steel plate with an APTES coating is an efficient substrate for imaging RHE tissue sections, (2) the thickness of the RHE tissue section impacts the number of lipid compounds detected, and (3) washing or recrystallization pretreatments can have a positive or negative impact on the signal intensity and the number lipid compounds identified.

Interestingly, there is currently no standard regarding the choice of substrate for the MALDI imaging of biological tissues despite this being a crucial step in the generation of ions (since the conductivity affects the ionization/transmission of ionized molecules, e.g., lipids). This observation was also made by Feucherolles and Frache during their analysis of microbiological applications.^54^ Various substrates have been used to analyze lipids in skin samples, including aluminum plates,^40^ gold-coated MALDI plates,^12^ as well as conventional microscope glass slides.^36−38^ Glass slides are mainly used so that histological staining, e.g., hematoxylin and eosin, can be performed in parallel to MALDI imaging. Of the different substrates used here, APTES-coated steel plates generally resulted in a better ion signal than conventional and ITO-coated glass slides. This is likely due to the combination of the inherent conductivity of the substrate and the improved adhesion provided by the APTES solution. The APTES coating-based solution increases the adhesion of frozen tissue sections to different substrates by modifying the surface of the substrate with a positive charge.^42^ Like aluminum, stainless-steel plates exhibit a high conductivity compared to materials like conventional or ITO-coated glass slides due to their bulk metallic property. However, one of the drawbacks of using a thin aluminum plate is its tendency to bend slightly, causing the substrate to deviate from a perfect plane and a shift in the laser (as observed in our studies, data not shown). The use of the steel plate alone increases conductivity but requires a coating to provide sufficient adhesion of the outer layers of the RHE tissues. This study indicated that coating the plates with APTES significantly improved the adhesion of the RHE tissue sections. Therefore, by combining the high conductivity of the more robust steel plates and the good adhesion afforded by APTES, APTES-coated steel plates could be used as a substrate for high-spatial-resolution analysis of RHE samples. Further investigations of the topography of the samples (e.g., adhesion of tissue sections and matrix layer morphology) are beyond the scope of this study.

The effect of the thickness of the RHE tissue section on the signal intensity and the number of lipid compounds was evaluated. Indeed, the preparation of the tissue sections is an important step for acquiring high-quality images, as reported by others.^55,56^ For example, Sugiura et al. investigated the impact of different thicknesses of brain and liver sections (2 to 40 μm) on protein profiles and observed that number of peaks and their related intensities increased in tissue sections thinner than 10 μm.^55^ Similarly, Yang and Caprioli evaluated the impact of the tissue thickness (1 to 16 μm) on protein profiles. In their study, the protein profiles in tissue sections ranging from 4 to 16 μm thick were identical.^56^ More recently, Wang et al. identified an optimal thickness for brain sections to be 2–6 μm.^57^ While these studies investigated proteins with different mass ranges, i.e., 3000 to 21 000 Da, there are few reports focusing on lipid profiles, despite the reported marked changes in lipid signal intensities between tissue sections of different thicknesses.^58^ Moreover, to the best of our knowledge, there are no reports highlighting the impact of the thickness of RHE tissue sections or *in vivo*/*ex vivo* skin slices on lipid signals. In the present study, RHE tissue sections between 4 and 10 μm were compared, which is within the range of thicknesses investigated previously for similar studies using human skin, i.e., 8 to 12 μm.^8^ The results indicated that more glycerophospholipid and sphingolipid matches were identified, and the intensity signal was greater for certain *m*/*z* values in the 4 μm thickness RHE tissue sections. Interestingly, 71 compounds were also specific to the 4 μm sections. Based on these parameters alone, it could be assumed that 4 μm thickness slices provide the most information and is thus the optimal thickness for RHE imaging; however, technically, this thickness is also the most difficult to prepare. Indeed, cryo-sectioning of CMC embedded RHE models to achieve thicknesses below 6 μm was challenging. Moreover and somewhat surprisingly, 5 μm tissue sections resulted in the poorest image quality of the thicknesses tested. Therefore, we recommend the use of 6 μm slices of RHE models, as these are technically easier to prepare and gave reasonable results for both intensity and the number of identified compounds.

Washing is regarded as a crucial pretreatment step when preparing samples for MALDI MSI analysis, since it eliminates endogenous salts and potential interference from contaminants such as residual embedding media, which may impede the desorption/ionization process.^59^ This is also the case for analyzing lipids, whereby washing with an aqueous solution is reported to increase the sensitivity in both ion modes.^8,43^ Some researchers used deionized water,^14^ while others reported that pH-adjusted aqueous solutions improved the detection of specific compounds.^50^ For example, Angel et al. showed that ammonium formate (pH 6.4) or ammonium acetate (pH 6.7) solutions significantly increased the signal intensity and number of analytes detected in adult mouse brain tissue sections.^43^ Others have compared the presence of lipids in artificial skin model samples prepared with and without a wash with deionized water.^8,38,40^ In the current study, we compared the effects of washing with either deionized water or NH_4_Ac. Washing tissues generally resulted in a higher signal intensity than unwashed tissues. There were also significantly more compounds detected in washed samples as well as *m*/*z* values that were absent in unwashed samples. There was little difference in the results using NH_4_Ac or Milli-Q water, with the exception that 50 compounds were specific to tissues washed with Milli-Q water. In addition, despite the lateral resolution used, i.e., 5 μm, and the average reduced size of RHE sample (∼500 × 200 μm), there was no major delocalization of the compounds, i.e., migration/diffusion across and away from the tissue. Likewise, others have reported that water washes do not appear to cause compound delocalization at a spatial resolution of 150 μm.^14^ The pipet washing protocol used in the present study, which was previously reported to lead to an undesired diffusion of molecules over the tissue surface,^60^ did not occur in the current study. Other alternative washing protocols are available, including immersion or wet paper-based tissue blotting.^60^ As the name suggests, the immersion method involves submerging the tissue sections in a bath of the selected wash solution and has been used for human skin samples washed in deionized water.^39^ The major disadvantage of this method for small RHE tissue sections is that they can easily detach from the substrate due to turbulence in the solution. The paper-based tissue washing method involves wetting a wipe with the wash solution and placing it on the top of the tissue section before being carefully removed.^60^ This method was not tested in this study to avoid any risk of delamination of the skin layers or detachment of the whole RHE tissue section from the substrate.

Matrix recrystallization is a process used in MALDI mass spectrometry imaging to improve the quality of the mass spectra obtained from biological tissues. In this process, the matrix used for sample preparation is dissolved and then allowed to recrystallize on the tissue surface in a controlled manner. This method has already been used for imaging proteins and lipids at high spatial resolution in chicken liver and maize, respectively.^45,56^ Interestingly, although this practice is commonly employed for biological samples, there are currently no reports regarding its application to skin samples. The recrystallization protocol performed in the present study was based on the method of Dueñas et al., who reported that recrystallization with 5% IPA at 55 °C for 2 min was optimal for lipid imaging. However, this IPA-based recrystallization did not improve further the signals in RHE tissue sections, which had already been washed. In some cases, recrystallization even decreased the intensity signal and the number of *m*/*z* values detected. Additionally, when the RHE tissue sections were subjected to recrystallization in a chamber with solvent vapors, the tissues tended to twist and partially or completely detach from the substrate. Overall, recrystallization of the HCCA matrix under the conditions used did not positively impact the final lipid intensity signal in RHE. Future studies could investigate whether recrystallization is more beneficial for more hydrophilic matrices, such as DHB, for RHE tissues.

There are several limitations to the current study. First, the matrix material, i.e., HCCA, and spray conditions, i.e., pneumatic sprayer, were fixed and not modified. This is important since the choice of the matrix and the method of application can affect the degree of delocalization of compounds.^61^ To image tissues at the cellular level, sample preparation should provide crystal sizes smaller than the diameter of the laser beam on the MALDI target.^56^ Others have used different matrices, e.g., 9-AA, HCCA, DHB, MBT, DAN, and SA for the imaging of *in vitro* and *in vivo* human skin as well as different types of matrix deposition methods, e.g., automatic sprayer, acoustic spotter, sublimation, or airbrush.^8^ A second limitation of the study was that other sample preparation steps which need to be considered were not specifically investigated, e.g., storage, the cryo-sectioning method, and the drying process.^50^ For example, while the temperature during sectioning can range between −10 to 30 °C, de Macedo et al. recommended that skin biopsies with significant subcutaneous tissue require colder temperature (−30 °C) due to the high content of lipids.^8^ Lastly, in the current study, sample drying was performed at atmospheric pressure for subsequent AP-MALDI imaging; however, others advocate the use of a vacuum approach, which might favor the diffusion of molecules in small size tissues, such as RHE models.^17^ Therefore, future investigations optimizing the RHE sample preparation for MALDI MSI could be used to evaluate these parameters.

## Conclusion

The development of increasingly sensitive hardware and computational methods has enabled researchers to achieve better resolution and quantification of components of biological samples at the cellular and molecular levels. However, high-quality data can be acquired only if the samples are prepared using an optimized workflow. This study highlights the impact of different substrates, sample thicknesses, wash methods, and matrix recrystallization on the analysis of lipids in RHE models. When considering the technically challenging aspect of cryo-sectioning, optimal and reproducible sample preparation resulting in significant signal intensity and numerous *m*/*z* values can achieved by a combination of (1) coated metallic substrates, such as APTES-coated stainless-steel plates, (2) RHE tissue sections with a thickness of 6 μm, and (3) washing with Milli-Q water before HCCA matrix spraying (without recrystallization). This refined methodology using AP-MALDI coupled to high-resolution mass spectrometer should improve the current sample preparation workflow to evaluate changes in skin composition after application of dermato-cosmetics.

## Supporting Information Available

Supplementary file 1 - TOF-SIMS analysis for the APTES coating (.docx). Supplementary file 2 - Peak list and peak identification of RHE with different thicknesses (.xlsx). Supplementary file 3 - Images of RHE tissue sections twisted during the matrix recrystallization process (.docx). Supplementary file 4 - Peak list and peak identification of RHE with different washings and recrystallization (.xlsx) (ZIP)
