# Supplementary material for: A Comprehensive Comparison of Tissue Processing Methods for High-Quality MALDI Imaging of Lipids in Reconstructed Human Epidermis
Source: J Am Soc Mass Spectrom. 2023 Oct 16;34(11):2469–80. doi: 10.1021/jasms.3c00185 (PMC10623569; doi:10.1021/jasms.3c00185)
Supplement: Supplementary file 1 — js3c00185_si_001.zip [file js3c00185_si_001.zip › SupplementaryFile_1_TOF-SIMS_Analysis.docx]

**Supplementary file 1: TOF-SIMS analysis**

**A comprehensive comparison of tissue processing methods for high-quality MALDI imaging of lipids in reconstructed human epidermis.**

Maureen Feucherolles1, *, William Le1, Jérôme Bour1, Carine Jacques2, Hélène Duplan2, Gilles Frache1, *

*1 Luxembourg Institute of Science and Technology (LIST), Molecular and Thermal Analysis, Materials Research and Technology, L-4422 Belvaux, Luxembourg.*

*2 Pierre Fabre Dermo-Cosmétique et Personal Care, Centre R&D Pierre Fabre, Avenue Hubert Curien, Cedex 01, 31025 Toulouse, France*

[**maureen.feucherolles@list.lu*](mailto:*maureen.feucherolles@list.lu) */* [*gilles.frache@list.lu*](mailto:gilles.frache@list.lu)

The experiments were performed using an IonTOF TOFSIMS 5 instrument, equipped with a bismuth cluster ion gun for analyses and imaging. Additionally, the instrument is fitted with a dual-source sputter gun (Cs^+^, C_60_^+^) for depth profiling and 3D imaging of materials.

Briefly, the technique relies on the use of a tightly focused primary ion gun (which provides a pulsed ion beam) to eject matter from the surface of the sample. Among the sputtered secondary particles, the sample emits electrons, neutral molecules/fragments, and elements but also a minor proportion of positively or negatively charged molecules/fragments and elements (called secondary ions). These secondary ions are analyzed by a Time of Flight mass spectrometer. The type of analyzer provides a high-speed measurement of the m/z ratio of the secondary ions emitted by the sample during each pulse of primary ion.

As the primary ion beam can be rastered across the sample (typically over 500 x 500 mm^2^), the individual spectra collected over this surface can be used for the retrospective image reconstruction of the lateral distribution of any secondary ion. In this study the instrument was operated in analysis and imaging mode, in using both positive and negative detection modes.


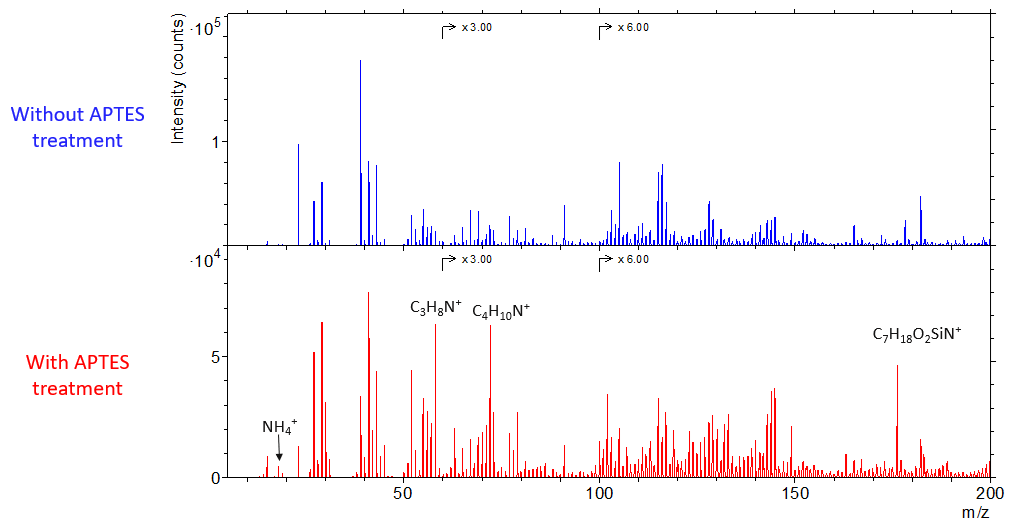


**Figure S1**. Detection of the ions NH_4_^+^, C_3_H_8_N^+^ and C_7_H_18_O_2_SiN^+^ ,fragment ions characteristic of gamma-aminopropyltriethoxysilane.


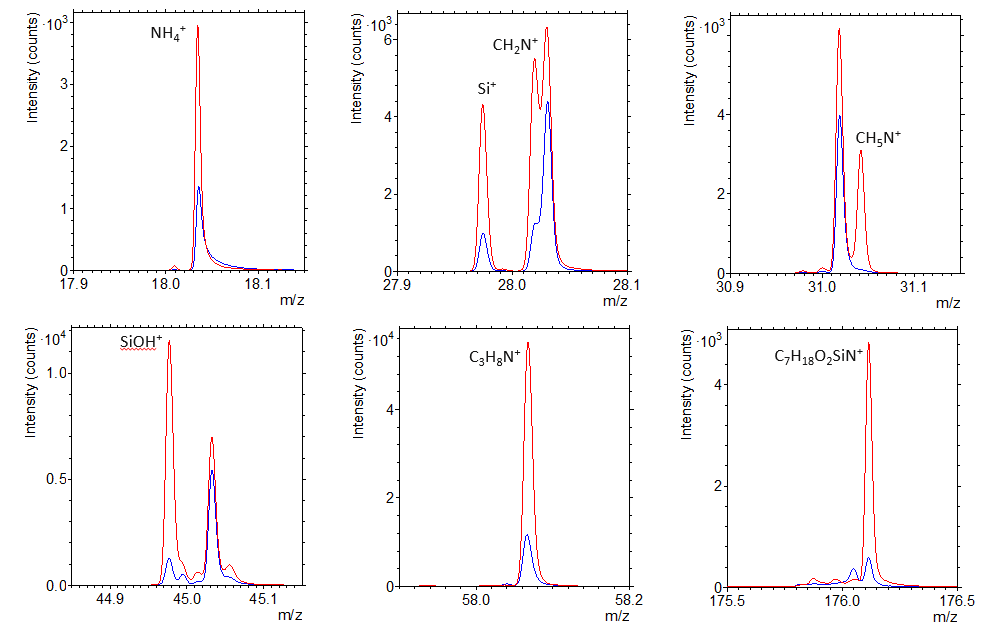


**Figure S2**. Detection of fragment ions characteristic of gamma-aminopropyltriethoxysilane. Blue: Without APTES treatment. Red: with APTES treatment
