# Supplementary material for: A Comprehensive Comparison of Tissue Processing Methods for High-Quality MALDI Imaging of Lipids in Reconstructed Human Epidermis
Source: J Am Soc Mass Spectrom. 2023 Oct 16;34(11):2469–80. doi: 10.1021/jasms.3c00185 (PMC10623569; doi:10.1021/jasms.3c00185)
Supplement: Supplementary file 1 — js3c00185_si_001.zip [file js3c00185_si_001.zip › SupplementaryFile_3_TwistedTissues.docx]

**Supplementary file 3: Tissues twisted because of the recrystallization**

**A comprehensive comparison of tissue processing methods for high-quality MALDI imaging of lipids in reconstructed human epidermis.**

Maureen Feucherolles1, *, William Le1, Jérôme Bour1, Carine Jacques2, Hélène Duplan2, Gilles Frache1, *

*1 Luxembourg Institute of Science and Technology (LIST), Molecular and Thermal Analysis, Materials Research and Technology, L-4422 Belvaux, Luxembourg.*

*2 Pierre Fabre Dermo-Cosmétique et Personal Care, Centre R&D Pierre Fabre, Avenue Hubert Curien, Cedex 01, 31025 Toulouse, France*

[**maureen.feucherolles@list.lu*](mailto:*maureen.feucherolles@list.lu) */* [*gilles.frache@list.lu*](mailto:gilles.frache@list.lu)

No Wash + Recrystallization


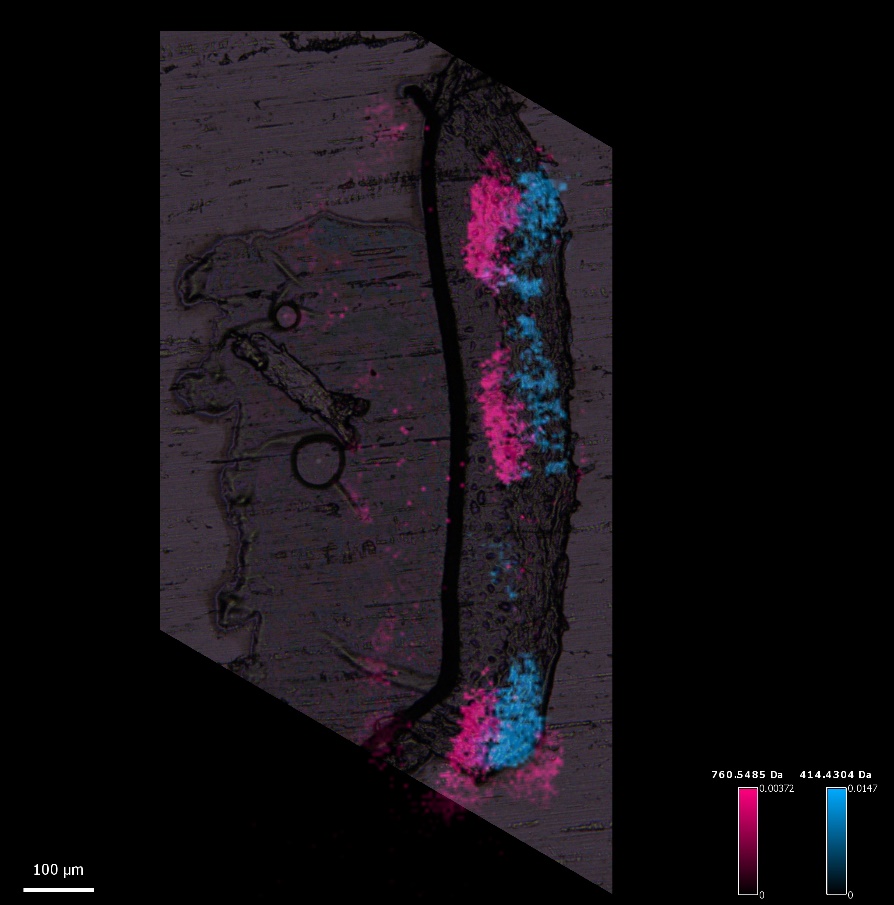


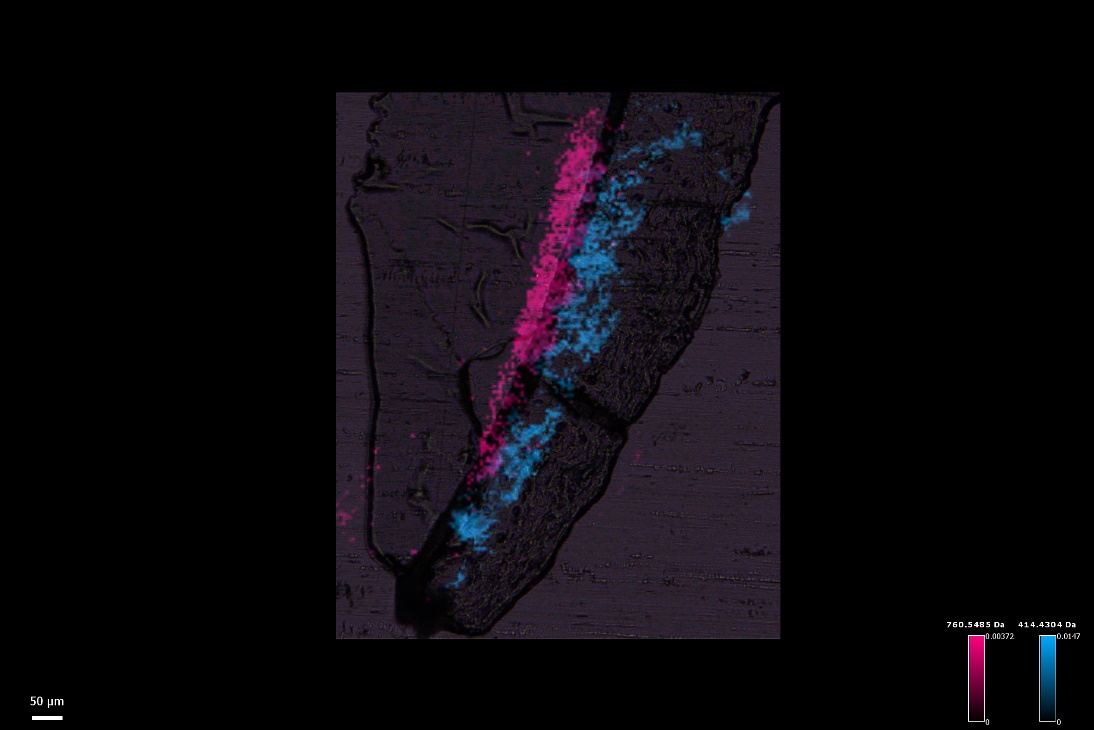
Water Wash + Recrystallization

The bright microscopy pictures were taken before the recrystallization. The overlaid MALDI images do not fit with the microscopy pictures due the twisted tissue after recrystallization.
